# Supplementary material for: Antiviral innate immunity is diminished in the upper respiratory tract of severe COVID-19 patients
Source: medRxiv. 2022 Nov 13:2022.11.08.22281846. Preprint. [Version 1] doi: 10.1101/2022.11.08.22281846 (PMC9681051; doi:10.1101/2022.11.08.22281846)
Supplement: Supplement 1 [file media-1.pdf]

Supplementary Figure 1

28 Type I Interferon Stimulated Genes	
EPSTI1	IFI44
IFIT1	IFI44L
IFIT2	IFI6
IFIT3	IFIT5
ISG15	LAMP3
LY6E	MX1
USP18	OAS1
CXCL10	OAS2
SOCS1	OAS3
GBP1	OASL
DDX60	RSAD2
HERC5	RTP4
HERC6	SIGLEC1

NF-kB enriched Score
AICDA
CCND2
EBI3
GZMB
IFNG
MSR1
SELL
SELP
TANK
TLR2
XIAP

IFNα and IFNγ regulated genes (orange: EPSTI1, IFIT1, IFIT2, IFIT3, ISG15, LY6E, USP18)

IFNα, IFNγ and NFκB1/2 regulated genes (dark grey: CXCL10, SOCS1)

IFNα and NFκB1/2 regulated genes (light grey: GBP1)

^Liu SY et al, PNAS, 2012

Figure S1. List of genes contained in the IFN I and NF-κB score. Calculation of an IFN score by 2 methods: a z-score-based standardized score and a geomean score. Calculation of an NF-κB score by 2 methods: a z-score-based standardized score and a geomean score

Supplementary Figure 2

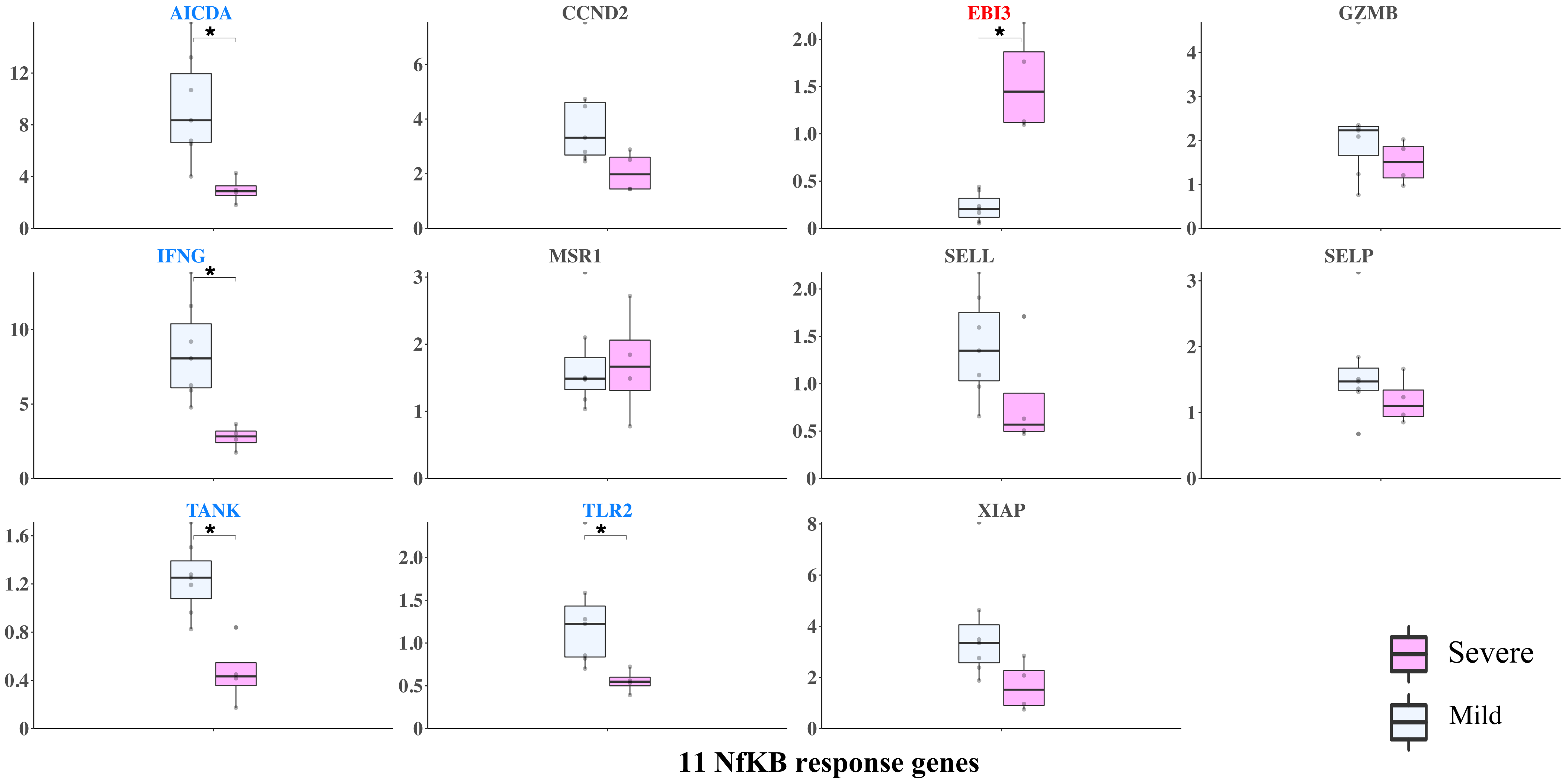

Figure S2. The 11 NF-κB response gene transcriptions were measured by NanoString in the upper respiratory tract (URT) of patients in the first ten days from the disease onset. mild (n=6 patients) and severe COVID (n=4 patients, the earliest sample is included). Data for both groups are shown as fold change from healthy controls. Blue labels indicate gene expression was significantly higher in the mild than in the severe cohort, Red label indicates gene expression was significantly higher in the severe than in mild cohort. *P<0.05, **P<0.01, ***P<0.001, ****P<0.0001.

Supplementary Figure 3

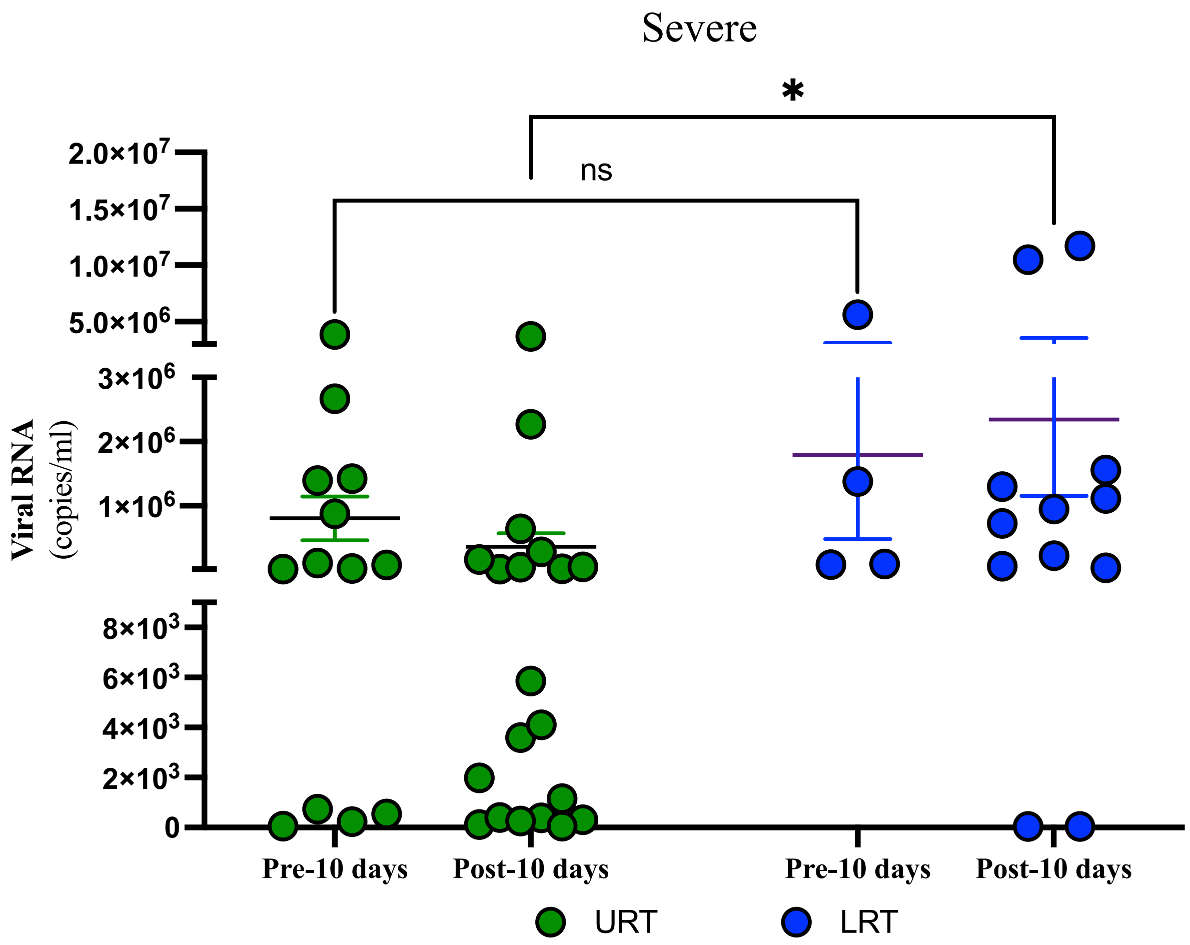

Figure S3. Viral load was measured by ddPCR from severe COVID-19 patients that had samples before and after 10 days post-symptoms onset. In the URT for the first 10 days, (n=4, 13 samples), post 10 days (n=4, 20 samples). In the LRT for the first 10 days (n=4, 4 samples), post 10 days (n=4, 12 samples). Individual dots indicate independent samples, solid line indicates the mean and SEM. 2-way Anova was used to compare between groups. Pre-10 days (Day 1 - Day 10), Post-10 days (Day 11-)

Supplementary Figure 4

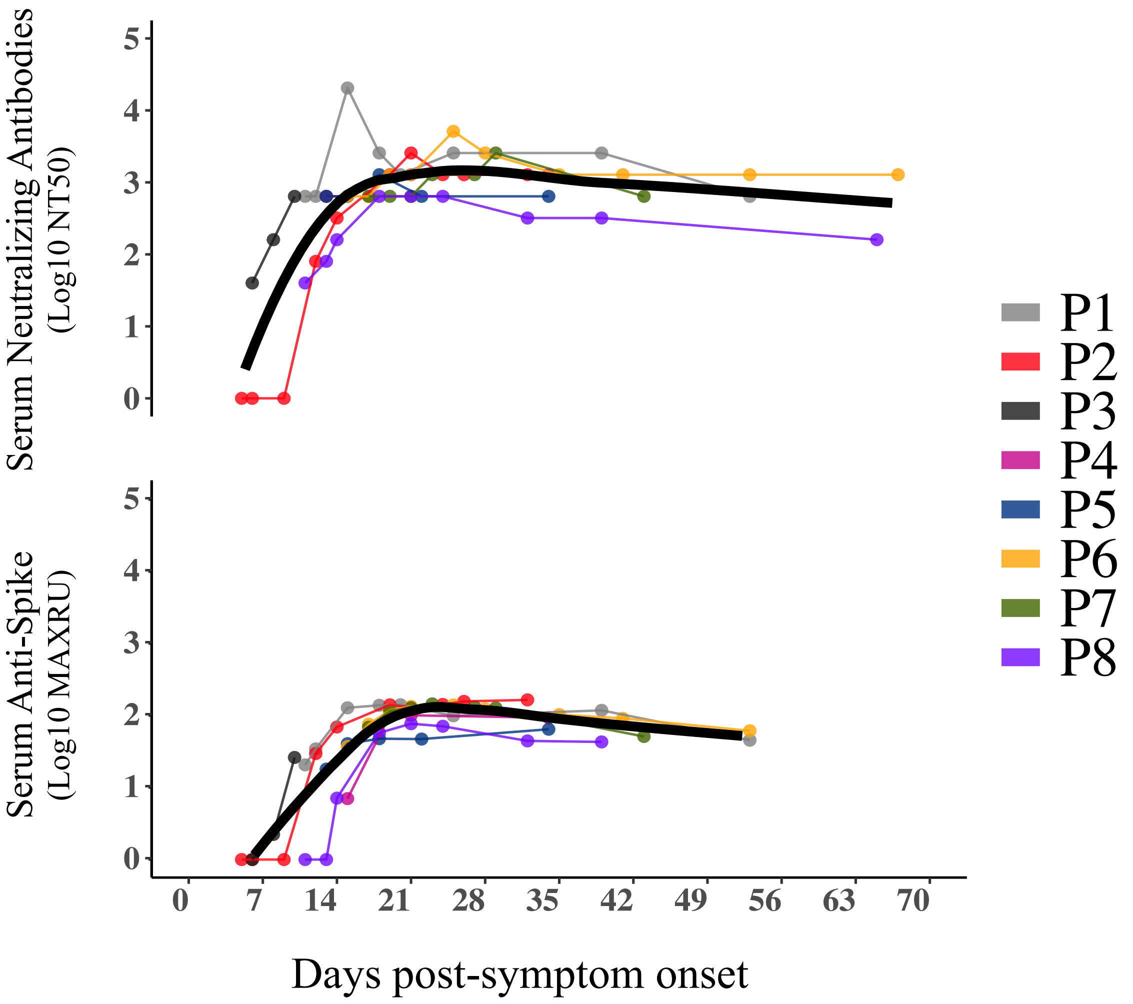

Figure S4. Serum Neutralizing Antibodies and Serum Anti-spike antibody levels in blood of severe COVID-19 patients. Data shown in logarithmic scale. Each patient is shown in different color. Black solid line shows the mean value.
